# Black Pastors’ Experiences of Occupational and Life Stress During COVID-19 in the USA

**DOI:** 10.1007/s10943-023-01901-9

**Published:** 2023-08-30

**Authors:** Robert C. Rogers, Taunya M. Tinsley

**Affiliations:** 1 Department of Counseling, Montclair State University, PO Box 1532, Morristown, NJ 07962-1532 USA; 2 Church of God in Christ, Morristown, NJ USA; 3 Pillar College, Newark, NJ USA; 4 Transitions Counseling Services, LLC, 63 Chestnut Road, Suite 10, Paoli, PA 19301 USA

**Keywords:** Occupational stress, Black pastor, Clergy stress, Black church

## Abstract

This study sought to identify the occupational stressors Black pastors experience, who serve in Black Church denominations and Black nondenominational churches. A total of 218 pastors completed the survey out of 2786 for a response rate of 10.1%. Black pastors identified their most challenging stressors as member dynamics, financial stress, leading a church to fulfill its mission, and pastor's workload. Black women pastors faced the additional stressor of having their pastoral leadership challenged by male congregants. Black pastors faced more stressors during the COVID-19 pandemic including church closures, transitioning to virtual services, unexpected deaths, and an increased workload with 72.5% of pastors reporting moderate to extreme stress levels. Approximately 77% of pastors acknowledged experiencing from moderate to extreme stress levels during social protests for the deaths of Black people by law enforcement. Black pastors further acknowledged experiencing an additional three to six life stressors outside of their pastoral roles.

## Introduction

The Black Church is the workplace setting where Black pastors experience stress while performing their spiritual and leadership responsibilities. As a religious institution, owned and operated by Black people, Lincoln and Mamiya (1990) described the Black Church as the “most important and dominant institutional phenomenon in African American communities” (p. 92). The term, Black Church, represents the variety of Black Christian faith communities, religious expressions, and spiritual practices by Black people in the USA (Douglas & Hopson, 2001; Gates, 2021; Mamiya, 2005). For example, there are many Black Church denominations, independent Black churches without a connection to an organized body of churches, and nondenominational churches who refuse to be labeled by a specific religious identity, such as Baptist, Methodist, or Pentecostal. The Conference of National Black Churches (CNBC), a consortium of eight historically Black denominations, reported that its membership represents 80% of Black Christians in the USA, which includes 30,000 churches and 15 million members (CNBC, n.d.). Regardless of the church name or affiliation, Black pastors have historically provided the leadership for Black churches (Gates, 2021; Mamiya, 2005).

The Black Church serves a pivotal role in helping families define the meaning of and strategies for Black existence in American society. The church helps to educate and empower Black congregants to live amidst adversity, changing family patterns, and denigration of their human dignity (Gates, 2021; Tinsley & Curtis, 2009). Black pastors have been serving in Black churches as religious and community leaders since the eighteenth century fostering spiritual, emotional, and mental wellness, facilitating community development, and empowering the survival of Black people in a racist America. As pillars of the church and community, Black pastors occupy a position of authority and influence as persons look to them for guidance in everyday matters and support in times of crisis (Harmon et al., 2018; Sklar & Goldman, 2023). Being in the public eye may be a source of stress for Black pastors.

The COVID-19 pandemic, with higher rates of infection and death hitting Black communities (Eligon et al., 2020) and government restrictions closing churches nationwide, brought serious challenges for Black pastors. DeSouza et al. (2021) acknowledged the historic impact of the closing of Black churches: “…for the first time in our history, African Americans must cope with the contextually valid fears of COVID-19 without physical access to our religious havens to alleviate mental distress” (p. 9). Black pastors faced challenges of leading their congregations during a pandemic without any training or preparation. While much attention was given to healthcare workers and first responders during the pandemic, less attention was given to Black pastors’ experiences of stress (Goldblum et al., 2023; Sklar & Goldman, 2023).

While dealing with the challenges of COVID-19, Black pastors continued to confront the pandemic of racial injustice during the public deaths of Black Americans by law enforcement and the resulting social protests (DeSouza et al., 2021). Black pastors have historically participated in social protests against racial injustice and provided support to persons impacted by different forms of racial microaggressions and White supremacist ideologies (Gates, 2021; Sue et al., 2019). Black pastors have provided emotional and spiritual support to persons experiencing racism-related life events and vicarious racism experiences as in the publicly viewed death of George Floyd. Black pastors' presence in the courtroom with the Ahmaud Arbery family so troubled the defense attorney that he sought to ban Black pastors from being there (Knowles, 2021). Just as racism-related stress can increase the stress burden on minority populations and negatively impact their mental health (Carter, 2007), Black pastors themselves are not exempt from experiencing racism-related stress and its impact on their well-being.

## Literature Review

Stress exists as an intrinsic element of the leadership role pastors hold and the work they perform (Adams et al., 2017; Darling et al., 2004; Hill et al., 2003). The occupational stress pastors experience results from the interpersonal dynamics, organizational structure, and responsibilities within their congregations where they serve as the “managers and executive directors” (Hulme, 1985, p. 11). Pastors' responsibilities include preaching and teaching, supervising staff, program planning, training leaders, counseling members, crisis intervention, and providing support during times of sickness and death (Adams et al., 2017; Carroll, 2006). Similar to emergency personnel, police, and social workers, pastors may experience schedule unpredictability, emotional exhaustion, and stress-induced physiological arousal (Adams et al., 2017). The stress pastors experience may affect them in various ways and in some cases can lead to burnout, nervous breakdowns, quitting being a pastor, and suicidal ideation (Doolittle, 2007, 2010; Turton & Francis, 2007).

Pastors may experience different sources of pastoral stress depending on the church size, surrounding community, and their dynamic interactions within these work environments (Lazarus & Folkman, 1984; Rogers, 2022b). Members may hold unrealistic expectations and demands upon pastors' time, availability, and resources, which may lead pastors to excessive busyness, efforts to keep everyone happy except themselves and their families, and overworking while sacrificing their own self-care (Berry et al., 2012; Chandler, 2010). Pastors may feel stressed by work overload where constant work demands never let up with ongoing deadlines and not seemingly enough time for the myriad of duties to be performed (Berry et al., 2012; Charlton et al., 2009). Both Wells et al. (2012) and Carroll (2006) reported how pastors' work responsibilities can impinge upon pastors' personal and family time, space, and activities creating boundary-related stress regardless of pastors' gender, race, and denomination. Pastors have experienced criticism of themselves and their families by church members, which has led pastors to feel dissatisfied with their jobs and congregations and have their physical and emotional health impacted (Carroll, 2006; Lee & Iverson-Gilbert, 2003). Pastors have acknowledged the financial stress from receiving low levels of compensation while Mamiya (2005) found that Black pastors received two-third of the compensation which White pastors received. Carroll (2006) further reported that 70% of Black pastors did not have a pension and 36% did not receive health benefits.

Researchers have documented various coping strategies pastors have used to combat stress and renew themselves. Pastors in a peer support group, which offered personal sharing, accountability, and support, experienced a reduction in psychological distress (McMinn et al., 2005; Miles & Proeschold-Bell, 2013). Spiritual practices, such as prayer, solitude, going on a retreat, journaling, and Bible reading, enhanced pastors’ spiritual wellness and contributed to higher feelings of personal accomplishment and lower feelings of emotional exhaustion and depersonalization (Chandler, 2010; Doolittle, 2010; McMinn et al., 2005). Social support, experienced through a network of supportive relationships in the family, congregation, community, and denomination, helped to combat depressive symptoms, emotional exhaustion, and anxiety symptoms while contributing to physical and emotional health, personal accomplishment, job satisfaction, and quality of life (Carroll, 2006; Doolittle, 2010; McMinn et al., 2005; Proeschold-Bell et al., 2015). Regular physical activity has enabled pastors to combat stress, burnout, hypertension, and depression (McMinn et al., 2005; Webb & Bopp, 2017).

The demands of congregational and public leadership may bring occupational and personal stress into Black pastors' lives. Ferguson (2007) stated, “Clergy are often in the unique position to be the first responders to individuals and families during times of trauma and crisis. …The very acts that get pastors rewarded in their ministry can also be the very things that wreak havoc on their family, personal, physical, and spiritual lives” (p. 16). Cooper (2019) reported the impact of stressors on several Black pastors in Indiana, which included symptoms of a depressive episode, feeling empty inside, and an anxiety attack which led to hospitalization. A Black Baptist church pastor in Virginia acknowledged to his congregation, the *Washington Post*, and *CBS This Morning Show* that he was exhausted and felt very distant from God (Bailey, 2019). Anderson (2022) presented Black pastors' first-hand accounts of dealing with pressure, pain, power, pride, being on a pedestal, and their efforts to persevere. Rogers (2022a) found that Black pastors experienced clergy distress once in a while, which Frenk et al. (2013) described as including challenges, criticism, excessive demands, loneliness, and isolation.

With this study’s focus on a Black population, the intersectionality of race needs acknowledgment, especially as extensive research has documented the resulting health disparities (Hicken et al., 2014; Williams, 2018). Sternthal et al. (2011) found that Black people experienced more stressors than Whites and Hispanics in such areas as acute life events, relationship stressors, community stressors, financial stressors, and early life stressors as well as a higher co-occurrence of two or more stressors than Whites. These stressors were associated with self-reported poor health and depressive symptoms while stressful life events, financial stressors, and relationship stressors were associated with a higher risk of chronic illnesses.

## Research Question

More research is needed to further identify and understand the types of stressors Black pastors experience. The purpose of this study was to fill the gaps in the literature, which has focused primarily on White, male pastors in White mainline Protestant denominations (Darling et al., 2004; Hill et al., 2003) by (1) identifying the stressors Black pastors experience, who serve in Black Protestant denominations and Black nondenominational churches, (2) understanding the church environment of the Black Church during the pandemic, and (3) highlighting the issue of clergy wellness for Black pastors. Specifically, the researchers sought to answer this question: What occupational stressors do Black pastors experience? The study was reviewed and approved by the University Institutional Review Board.

## Method

### Sample

Black pastors, serving as either senior pastors or co-pastors in Black churches in Black denominations and nondenominational Black churches, comprised the sample. An a priori power analysis using G*Power with an alpha level of 0.05, a minimum power of 0.80, and a moderate effect size of 0.15 determined a minimum sample size of 92 pastors. Two hundred and eighty-three pastors took the survey out of 2786 who received the survey, which equaled a response rate of 10.1%. The survey completion rate equaled 77% where 218 pastors out of 283 completed the survey. Informed consent was received from all participants.

The sample included 135 males (61.9%) and 83 females (38.1%); 53.2% were 45–64 years old and 34.4% 65 years and older; 72% served full-time and 28% part-time. The sample consisted of married (70.6%), single (15.1%), and divorced (10.6%) participants with a significant difference in marital status between male and female pastors. For male pastors, 81.5% were married, 8.1% single, and 8.1% divorced; for female pastors, 53.0% were married, 26.5% single, and 14.5% divorced. The majority of Black pastors (75.2%) worked in small churches (1–100 members), 19.3% in medium churches (101–350 members), and 5.5% in large churches (351+ members; Rogers, 2022a). See Table 1 for participant demographics.

**Table 1:** Participant demographic characteristics

	*N*	%
Gender		
Male	135	61.9%
Female	83	38.1%
Age		
18–34 years	7	3.2%
35–44 years	18	8.3%
45–64 years	116	53.2%
65 years and older	75	34.4%
Marital status		
Married	154	70.6%
Single	33	15.1%
Divorced	23	10.6%
Widowed	3	1.4%
Separated	3	1.4%
Prefer not to answer	2	0.9%
Denomination		
Methodist	58	26.6%
Church of God in Christ	57	26.1%
Baptist	35	16.1%
Pentecostal	26	11.9%
Independent/nondenominational	25	11.5%
Holiness/apostolic	10	4.6%
Other	7	3.2%
Employment status		
Full-time senior pastor	146	67.0%
Full-time co-pastor	11	5.0%
Part-time senior pastor	42	19.3%
Part-time co-pastor	19	8.7%

### Data Collection

Convenience sampling helped to reach Black pastors in Black denominations through the survey flyer being shared through the following denominations: African American Episcopal Church, African American Episcopal Zion Church, Apostolic Assemblies of Christ, Christian Methodist Episcopal Church, Church Of God In Christ, Full Gospel Baptist Church Fellowship, National Association of the Church of God, National Baptist Convention of America, National Baptist Convention USA, National Missionary Baptist Convention of America, and National Missionary Baptist Convention. Black pastors on LinkedIn and Facebook received survey flyers and a video requesting their participation over an eight-week period.

### Instrumentation

The questions used here were supplemental to the Clergy Occupational Distress Index, which focused on the stressors of excessive demands, personal criticism, loneliness, isolation, and challenges (Frenk et al., 2013). These questions allowed participants to respond directly to the research question by naming their specific stressors: (1) What has been or is one of the most challenging stressors for you in being a Black pastor serving in a Black church? (2) What was one of the most challenging stressors you experienced specific to and during the coronavirus (COVID-19) pandemic? (3) What was your level of stress during the COVID-19 pandemic? (4) What was your level of stress during the deaths of Black people by law enforcement (for example, George Floyd and Breonna Taylor) and the resulting social protests? (5) In addition to any stress you may be experiencing in your role as a Pastor, in how many other areas of your life are you experiencing stress, such as in money matters, family responsibilities, personal health, working on a degree, being a caregiver, personal relationships, death of a loved one, racial discrimination, neighborhood stressors (lack of safety, crime, violence), etc.?

### Data Analysis

For the first two questions, NVivo qualitative data analysis software was used to analyze and code the responses. Saldana (2016) described In Vivo coding as using “words or short phrases from the participants’ own language in the data record as codes” (p. 295), which was the coding methodology used. For the first question on the most challenging stressor as a Black pastor, 18 initial codes were combined into five stressor categories: member dynamics, financial stress, being a female pastor, pastor's workload, and leading the church to fulfill its mission. For the second question on the most challenging stressor specific the COVD-19 pandemic, 20 initial codes were combined into these five categories: church operations, congregational life, impact of the virus, bereavement and grief, and pastor’s workload (Rogers, 2022b).

For the remaining three questions on Black pastors' stress levels and the number of other prime areas of stress, descriptive statistics were first reported. Then the Mann–Whitney *U* Test tested for any differences between males and females on stress levels and number of additional stressor areas in life. The Kruskal–Wallis Test compared the scores on these questions between groups of Black pastors based on age range, years as pastor, church size, and denomination.

## Results

### Most Challenging Stressors for Black Pastors

#### Member Dynamics

Member dynamics included Black pastors' perceptions of church members' behaviors, which was the most noted stressor. Pastors acknowledged the lack of participation by church members, especially those members younger than 60 years. Pastors further acknowledged the large need for willing workers, faithful support, and consistent involvement for churches to operate effectively. Black pastors experienced stress when members and boards resisted new ideas and programs while holding onto past church traditions and history. One Black pastor acknowledged church members “wanting to remain in a status quo situation…and not wanting to progress into the 21st-century but wanting to keep the church in the organization the way they are most comfortable.” Black pastors also experienced stress when their churches exhibited a lack of organizational and spiritual unity. Many pastors invested significant time to promote unity, however, with little success. Some pastors attributed their churches' lack of unity to the lack of spiritual maturity among both members and church leaders.

### Being a Woman Pastor

Many Black women pastors described their most challenging stressor as being a woman in an historically male role in the Black Church where male church members intentionally and unintentionally undermined their pastoral authority and leadership. These experiences seemed to make female pastors feel uncomfortable, annoyed, frustrated, and even angry. These pastors also acknowledged criticism, resistance to change, and some members and leaders “not following the (pastor's) vision of the house but trying to create their own.” Black women pastors further experienced stress when compared to Black male pastors, who may have been their predecessors or contemporaries, and expected to emulate these male figures in their pastoral leadership styles and, thus, having their unique identity as women pastors invalidated. One participant keenly described the stress of being a Black woman pastor this way: “So often congregants forget that (women) pastors are human, have families, personal problems, challenges that men do not have. Their families are often put on hold so they can deal with the problems of those in the congregation. We are criticized for almost anything, we do not have the support of male pastors, have to preach through our pain, we cannot cry or we are judged to be too emotional, cannot express anger or we are judged to be over the top.”

### Financial Stress

Black pastors acknowledged that financial stress manifested in two forms: the lack of church financial resources and the amount of financial obligations to their denominations. Black churches rely primarily upon members giving tithes, which equals 10% of their weekly paycheck, and offerings, voluntary amounts given to the church's general operating expenses or specific programs, for the church to be financially solvent. With the mandatory church closures during the pandemic, Black pastors implemented online giving platforms, such as Givelify, CashApp, Zelle, and PayPal, in order to continue to receive operating funds. Members, who chose not to use these online platforms, could mail their contributions to their church.

The lack of financial resources seemed to have its source in a small church size, socioeconomic status of members, and lack of stewardship and tithing. This lack of operating funds placed a financial burden on both the church and pastor. For the church, this meant not having enough financial support for operational expenses and programs. This presented challenges to address needed repairs and building maintenance costs for both problems already known and those which appeared unexpectedly, especially if the church building was very old. For the pastors, the lack of church financial resources meant not receiving an adequate salary or no salary at all.

Churches in a hierarchical denominational structure, like the African Methodist Episcopal, African Methodist Episcopal Zion, Christian Methodist Episcopal, Church Of God In Christ, are required to pay financial assessments to their denominational headquarters. Meeting these financial requirements was a major source of stress, especially when the church had declining attendance and aging membership, limited financial resources, and when these assessments were increased. Pastors described this stressor as including (1) the strong expectation and pressure that the financial assessment will be paid on time even if paid out of the pastor's personal funds and (2) the potential consequences of the pastor and church losing standing with the denominational leadership and, in the Methodist denominations, the pastor being moved to a different or lower status church if the assessment was not paid.

### Leading Church to Fulfill Its Mission

Black pastors acknowledged the stress in members responding to their leadership to fulfill the church's mission, particularly in achieving growth in membership. Black pastors faced the challenge of broadening members’ understanding of the church’s mission, which included making a paradigm shift of the church being private for members only to being open, inviting, and welcoming to non-church members. Black pastors identified challenges of helping members to use their gifts and talents to further church growth, finding ways to attract youth and young adults especially in churches with an aging population, and motivating the congregation to reach outside the church to focus more on community outreach and service.

### Pastor's Workload

Black pastors identified their workload as a major stressor, especially when members consistently looked to them as the resource person for information, guidance, and needs being met as well as being on-call 24/7. Pastors further expressed frustration when their workload increased and “no one offers any suggestions or help.” One female pastor described this stressor as “finding competent, available and willing persons to support the work of the ministry and assist me in the operation of the church.” The stress of the pastor's workload seemed to be exacerbated for pastors who worked a secular job. With the burden of other full-time work responsibilities, this challenged them with finding some measure of secular job/church work/personal life balance. Several Black pastors described weekly preaching preparation and delivery with relevance, creativity, and effectiveness as a major stressor. Black pastors also acknowledged experiencing loneliness and isolation as integral parts of their workload with church and community members not understanding all that pastors do and placing them on an unrealistic pedestal of perfection and superhuman qualities.

### Pastoral Stress During the COVID-19 Pandemic

Since this study was conducted during the COVID-19 pandemic, it was important to acknowledge and investigate the stress-related impact on Black pastors. Table 2 depicts the levels of stress Black pastors experienced during the pandemic. Black pastors faced these specific challenges: (1) moving to online worship services and Bible study, (2) maintaining contact with members they did not see in-person, (3) providing information to overcome skepticism and fears of the vaccine, (4) facilitating members' connection to prevent isolation, especially for seniors living alone, (5) keeping the church operating, and (6) grieving the deaths of family and church members, clergy colleagues, and close friends (Rogers, 2022b).

**Table 2:** Level of stress during COVID-19 pandemic

Level of stress	Male		Female		Total	
	*N*	%	*N*	%	*N*	%
Not at all stressful	11	8.1%	6	7.2%	17	7.8%
Slightly stressful	30	22.2%	13	15.7%	43	19.7%
Moderately stressful	45	33.3%	28	33.7%	73	33.5%
Very stressful	36	26.7%	26	31.3%	62	28.4%
Extremely stressful	13	9.6%	10	12.0%	23	10.6%
	135	100.0%	83	100.0%	218	100.0%

A Mann–Whitney *U* test revealed no significant difference in COVID-19 stress levels between Black female (Md = 3/moderately stressful, *n* = 83) and male pastors (Md = 3/moderately stressful, *n* = 135), *U* = 6138, *z* = 1.228, *p* = 0.22, *r* = 0.00. Kruskal–Wallis tests revealed no statistically significant difference in COVID-19 stress levels for Black pastors based on age range (*c*^2^ (3, *n* = 218) = 5.47, *p* = 0.14), years as pastor (*c*^2^ (2, *n* = 218) = 2.67, *p* = 0.263), church size (*c*^2^ (2, *n* = 218) = 4.40, *p* = 0.111), and denomination (*c*^2^ (6, *n* = 218) = 4.18, *p* = 0.653)

#### Transition to Virtual Services

One of the most challenging stressors for Black pastors was the closing of the church building, which created the inability to worship in-person, connect with other members, and members receiving emotional and spiritual support from each other. For some pastors, their congregations were closed for a year or more, which was a source of chronic stress. COVID-19 forced the transition to online worship services, which proved challenging to pastors and church members with little or no experience being online and using both technology and social media. This required pastors to learn online and social media platforms and getting members accustomed to using this technology. One pastor expressed his stress in “persuading people that there is a new normal and trying to persuade people to accept change.” Some pastors expressed concerns with the “lack of technical expertise and reliable internet service” to make this transition. Other pastors experienced stress from (1) not knowing who would attend on-line services, (2) learning to preach and teach to a computer screen without immediate feedback from members, and (3) not being sure the technology was working properly.

#### Bereavement and Grief

The pandemic confronted pastors and congregations with the reality of the unexpected deaths of members and loved ones, all of which created shock waves within congregations. One pastor lamented about “losing 60+ members during the pandemic” while other pastors expressed similar grief over the death of church members, leaders, and relatives of church members. Pastors also experienced stress in conducting funeral services within pandemic restrictions of closed caskets and attendance limits, which did not bring sufficient comfort and closure to the families. These restrictions prevented congregations from having their traditional homegoing funeral services, which celebrated the lives and contributions of the deceased. Pastors also faced the deaths of their own family members, such as one pastor who lost “15 people that are close to me” and another pastor who “lost 5 family members and several friends.” Even with losing family and friends, pastors reported serving their congregations untiringly.

#### Pastor's Increased Workload

Black pastors reported both an increased workload and added stress to their already busy roles. One pastor acknowledged the burden of seeing that the congregation had its basic needs met with families impacted by job closings and loss of income. One female pastor also acknowledged "carrying the church on my shoulders while struggling to keep communication lines open and worship engaging." Several pastors reported contracting COVID-19 with varying degrees of seriousness including being hospitalized and out of commission for several weeks. Even with contracting the virus, they were still pastors of congregations where one pastor stated, “trying to spiritually keep the congregation together.” Another pastor commented, “I was hospitalized with COVID-19 (before the vaccine was available) and I had to maintain a level of maturity with the leadership team, my family and the church.” These challenges raised the issue of self-care for pastors needing to find church work/personal life balance, which led some pastors to express concern of unrealistic expectations by church members. In one case, a pastor had COVID-19 and was yet “expected to serve others” while a female pastor “was taking care of church needs and being a caregiver for my mother.”

### Other Areas of Stress for Black Pastors

In addition to dealing with the COVID-19 pandemic, the country confronted the death of Black people by law enforcement, which resulted in massive social protests. Black pastors acknowledged experiencing stress during these social protests with their level of stress ranging from “not at all stressful” to “extremely stressful.” Over half (55.1%) of Black pastors reported being “very stressful” and “extremely stressful” while 22% acknowledged being “moderately stressful.” See Table 3 for a breakdown of responses.

**Table 3:** Level of stress during social protests

Level of stress	Male		Female		Total	
	*N*	%	*N*	%	*N*	%
Not at all stressful	6	4.4%	4	4.8%	10	4.6%
Slightly stressful	25	18.5%	15	18.1%	40	18.3%
Moderately stressful	35	25.9%	13	15.7%	48	22.0%
Very stressful	39	28.9%	29	34.9%	68	31.2%
Extremely stressful	30	22.2%	22	26.5%	52	23.9%
	135	100.0%	83	100.0%	218	100.0%

A Mann–Whitney *U* test revealed no significant difference in social protest stress levels between Black female (Md = 4/very stressful, *n* = 83) and male pastors (Md = 4/very stressful, *n* = 135), *U* = 6039.5, *z* = .997, *p* = 0.319, *r* = 0.00. Kruskal–Wallis tests revealed no statistically significant difference in COVID-19 stress levels for Black pastors based on age range (*c*^2^ (3, *n* = 218) = 1.00, *p* = 0.799), years as pastor (*c*^2^ (2, *n* = 218) = 0.17, *p* = 0.918), church size (*c*^2^ (2, *n* = 218) = 3.97, *p* = 0.138), and denomination (*c*^2^ (6, *n* = 218) = 4.60, *p* = 0.596)

Black pastors reported experiencing stress in several other areas of life in addition to the stressors experienced in their occupational roles. Pastors cited examples of family members being sick, caring for a dying mother, working a secular job, going to school, financial problems, moving to a new city, marital conflict and separation, and community stress of “young men killing each other.” Responses ranged from zero to six with pastors identifying three areas as the mode and median in their responses. See Table 4 for these responses.

**Table 4:** Number of other areas of life with stress

	Male		Female		Total	
	N	%	N	%	N	%
0	3	2.2%	5	6.0%	8	3.7%
1	18	13.3%	3	3.6%	21	9.6%
2	16	11.9%	18	21.7%	34	15.6%
3	46	34.1%	20	24.1%	66	30.3%
4	26	19.3%	16	19.3%	42	19.3%
5	13	9.6%	12	14.5%	25	11.5%
6	13	9.6%	9	10.8%	22	10.1%
	135	100.0%	83	100.0%	218	100.0%

A Mann–Whitney *U* test revealed no significant difference in the number of other areas of life with stress between Black female (Md = 3 areas, *n* = 83) and male pastors (Md = 3 areas, *n* = 135), *U* = 5844, *z* = 0.546, *p* = 0.585, *r* = 0.00. Kruskal–Wallis tests revealed no statistically significant difference in number of other areas of life with stress for Black pastors based on age range (*X*^2^ (3, *n* = 218) = 2.06, *p* = 0.56), years as pastor (*X*^2^ (2, *n* = 218) = 1.22, *p* = 0.543), church size (*X*^2^ (2, *n* = 218) = 2.02, *p* = 0.365), and denomination (*X*^2^ (6, *n* = 218) = 2.08, *p* = 0.912)

## Discussion

This study intended to identify and understand Black pastors’ experiences of occupational and life stress and their stress during the COVID-19 pandemic and racial justice protests in the USA. The participants named specific stress points associated within their pastoral service and personal lives. Consistent with past research, it is important to note that “clergy work-related poor psychological health, stress, and burnout pose an increasingly serious problem” (Lewis et al., 2007, p. 2). The participants continued the practice of putting the congregation’s needs and wellness before their own, striving to help members survive COVID-19, and cope with racism-related stress. Black church members also continued to look to their pastors as first responders in times of crisis (Goldblum et al., 2023; Sklar & Goldman, 2023).

This study unearthed the sentiments of Black female pastors who described the complexity of being a woman serving in an historically male role within a Black church. They consistently spoke of male members as intentionally and unintentionally undermining their pastoral leadership. Black women pastors specifically named examples of gender-related microaggressions that contributed to feelings of being uncomfortable, annoyed, frustrated, and angry. These results supported Miles and Proeschold-Bell (2013) and Carroll (2006) who found that women pastors reported more gender-based disparities than their male counterparts and reported different congregational expectations, pressures, and constraints.

The stressors Black pastors experienced during the COVID-19 pandemic included leaving the traditional brick and mortar structures and transitioning to virtual environments. Having to manage the large number of unexpected deaths of members and loved ones increased Black pastors’ levels of grief and loss, mourning, and vicarious trauma. In addition, an increase in Black pastors’ workload during the pandemic added more tension to their already demanding roles. Our study results also highlighted Black pastors’ additional stressful life experiences of being overwhelmed with caregiving responsibilities for sick and dying family members, working a secular job, matriculating through school, thwarting financial instabilities, relocation to a new city, marital separation or conflict, and community violence.

Previous research has shown that pastoral responsibilities and public leadership may bring occupational and personal stress that may lead to negative physical and mental health (Darling et al., 2004; Hill et al., 2003). These life stressors combined with occupational stressors raise the question of whether Black pastors realize their real level of stress and what coping strategies, if any, they employ. It is important that Black pastors significantly increase their awareness of symptoms of stress, compassion fatigue, and vicarious trauma and learn effective coping strategies to manage their stress levels.

Like their predecessors during the Civil Rights Movement, Black pastors demonstrated public leadership and championed social justice during the social protests following the deaths of Black people by law enforcement (Gates, 2021). With these deaths in public discourse, 95.4% of Black pastors acknowledged experiencing slight to extreme stress stemming from these social protests. These stress levels, while experienced by the participants, may not be known by church and community members who look to their pastors for leadership, inspiration, and meaning making in times of crisis. Likewise, pastors themselves may not have been aware of their stress levels and its impact on them and their congregants during these public upheavals.

## Implications

Studies have highlighted that pastors often face both life and occupational stress that can lead to mental health distress (Barna Group, 2017; Miles & Proeschold-Bell, 2013). The literature highlights a strong relationship between depression and physical health, critical congregants, social isolation, personal condemnation, boundary ambiguity, familial disapproval, and financial stress (Hill et al., 2003; Miles & Proeschold-Bell, 2013). Without effective coping strategies, Black pastors are at risk for negative consequences impacting their mental, emotional, and physical health. This highlights the necessity for support and coping strategies that effectively promote mental health and emotional wellness (Barna Group, 2017).

Black pastors identified some of their major stressors resulting from member dynamics. Black women pastors have the additional burden of responsibility to address the invalidation of their pastoral identity and authority. Black pastors must find proactive ways to address these dynamics in vivo and over time with individuals, boards and committees, and the entire congregation to lessen their stress and foster a healthier church environment. Black pastors must lead by example in their interactions and public discourse to model healthy relationships, boundaries, and organizational functioning that may assist to reduce their stressors.

It is important for Black churches to foster an environment supportive of Black pastors seeking help for occupational and life stress, mental health, and well-being. Black pastors and Black churches may benefit from collaborating with mental health professionals, pastoral counselors, and theological institutions who are aware of the importance of attending to Black pastors’ health and well-being. It is also vital that mental health practitioners and the community-at-large are trauma-informed and address the effects of generations of enslavement, oppression, microaggressions, and other race-based traumatic experiences on African descendants that may assist with coping and surviving generational trauma manifested today (Carter, 2007; DeGruy-Leary, 2017). It is further important to build upon the spiritual strengths, emotional resiliency, and determination that have empowered Black people and Black pastors, as they learn to heal from psychological injuries and challenging stressors.

Furthermore, it will be important for churches and communities to diminish stigmas and negative stereotypes on seeking counseling services through education about counseling services, stressors, mental health, and wellness. This may change beliefs positively to encourage Black pastors as well as church members to seek counseling services. The task for mental health professionals is to understand the circumstances which Black pastors work under and be spiritually competent to provide effective support, therapeutic strategies, psychoeducation, and clergy self-care training.

While theological institutions seek to train pastors, the stressors Black pastors experience highlights the need for theological institutions to expand their training to prepare pastors to accurately appraise stressful encounters, implement effective coping and self-care strategies, and transform stress-related congregational dynamics. No pastors were trained in church leadership for a pandemic, which now warrants theological institutions to include lessons learned from pastors in their curriculum. With our findings indicating the impact of mental and emotional stressors from racial, gender, and social justice incidents, it behooves theological institutions to integrate multicultural and social justice competencies (Ratts et al., 2016) in their work with pastors. Walker-Barnes (2023) encouraged “clergy, seminary faculty, faith-based activists, and other Christian leaders to view self-care as a strategy for sustaining the work that God has called them to do” (p. 20) for which she teaches a graduate course on self-care at her seminary in the hope that helping seminarians develop healthy practices now will reduce the risk of ministry burnout in the years to come. Another example is Pillar College’s pastoral self-care course in their Master’s in Ministry Leadership Program that focuses on (a) the theological and psychological perspectives of self-care, soul care, wellness, and mental health, (b) the natural consequences of helping (stress, burnout, loss and grief, compassion fatigue, vicarious trauma, etc.), (c) self-care programming and strategies for ministry leadership, and (d) research regarding the prevalence of these conditions in the fields of helping and ministry (Tinsley, 2023). It would behoove other theological institutions to follow these examples and provide required trainings and courses for future pastors to take seriously this subject matter, which seeks to prepare them for the realities of being a pastor.

Through the leadership of Black pastors, Black churches have historically supported the wellness, empowerment, and survival of Black people. This study focused on the impact of a pandemic and racial justice protests. Yet, there are other existential crises impacting Black churches and communities, such as the rise in Black youth suicide, Black maternal health, adverse childhood experiences, and the developmental needs of younger Black generations who may not embrace the traditional Black Church faith experience (Pew, 2021), which may challenge Black pastors' ability to respond to these problems and impact their stress levels. Additionally, Black denominations may not realize how their denominational structures or practices (e.g., financial assessments, budgets, church politics, etc.) may contribute to Black pastors’ stress. Black denominations may want to examine these dynamics to reduce Black pastors' stressors and increase their mental, emotional, and physical health.

## Limitations

There are a few limitations to this study that should be mentioned. First, this study relied on convenience sampling rather than random sampling, which limited the representation of Black pastors, omitted pastors from some denominations, and may have restricted the sample size. Second, the open-ended questions relied on self-reported responses by participants rather than through qualitative interviews, which resulted in no fact or member checking by the researchers and possible socially desirable responding by participants. Third, the length of the data collection instruments may have prevented some participants from responding more fully to the open-ended questions due to their time constraints. Fourth, the study was conducted during the COVID-19 pandemic, whose environment may have presented a bias in participants' responses. Finally, the majority of the sample were pastors of small churches whose responses may have overshadowed the voices of pastors in medium and large churches.

## Suggestions for Future Research

There are a few areas in which future research may be helpful. First, a further identification of the stressors Black pastors experience along with trends and patterns would be advantageous to provide a more in-depth analysis of their experiences. Second, Black female pastors have some different experiences from Black male pastors, which need further investigation through both qualitative and quantitative research. For example, what may be some influencing factors that contribute to Black women pastors to be more likely single and divorced than Black male pastors? Third, while the Caribbean-descended and immigrant groups may be 10–15% of the US Black population (Neighbors et al., 2007), future research may want to study Black pastors of different ethnic origins because of the contribution of culture to population group differences. Fourth, since Black pastors in this study experienced on average the co-occurrence of three stressors in addition to pastoral stressors, future research may investigate the relationship of these additional stressors to self-reported health symptoms and chronic illnesses. Finally, future research can examine the coping strategies Black pastors employ to combat their stressors and contribute to their wellness.

## Conclusion

Black pastors identified their most challenging occupational stressors as member dynamics, financial stress, leading a church to fulfill its mission, and pastor's workload. Black women pastors faced the additional stressor of having their pastoral identity and authority challenged by male congregants who often compared female pastors to male predecessors or contemporaries. Black pastors faced more stressors during the COVID-19 pandemic including church closures, transitioning to virtual services, the unexpected death of members, family, and friends, and an increased workload with 72.5% describing their stress level ranging from moderately stressful to extremely stressful. Approximately 77% of pastors acknowledged experiencing from moderate to extreme stress levels during social protests resulting from the deaths of Black people by law enforcement. Black pastors further acknowledged experiencing an additional three to six life stressors outside of their pastoral roles. This study fills a gap in the literature by identifying the occupational and life stressors Black pastors experience.
